# Metabolic modeling predicts synergistic growth benefits between arbuscular mycorrhizal fungi and theoretical N_2_-fixing rhizobia symbiosis in maize

**DOI:** 10.1093/plphys/kiag327

**Published:** 2026-06-02

**Authors:** Joshua A M Kaste, Rourou Ji, Patrick Sydow, Ruairidh J H Sawers, Megan L Matthews

**Affiliations:** Department of Civil and Environmental Engineering, Grainger College of Engineering, University of Illinois Urbana-Champaign, 205 N Mathews Ave., Urbana, IL 61801, United States; Department of Civil and Environmental Engineering, Grainger College of Engineering, University of Illinois Urbana-Champaign, 205 N Mathews Ave., Urbana, IL 61801, United States; Department of Plant Science, College of Agricultural Sciences, The Pennsylvania State University, 102 Tyson Building, University Park, PA 16802, United States; Department of Plant Science, College of Agricultural Sciences, The Pennsylvania State University, 102 Tyson Building, University Park, PA 16802, United States; Department of Civil and Environmental Engineering, Grainger College of Engineering, University of Illinois Urbana-Champaign, 205 N Mathews Ave., Urbana, IL 61801, United States; Carl R. Woese Institute for Genomic Biology, University of Illinois Urbana-Champaign, Urbana, IL 61801, United States

## Abstract

Engineering a novel N_2_-fixing rhizobia symbiosis in cereal crops is a strategy being pursued to improve agricultural sustainability. However, if such a symbiosis were introduced, it would have to be economically viable in the context of plants’ existing nutrient acquisition strategies, including existing symbioses with arbuscular mycorrhizal fungi (AMF) that most plants already engage in. It is important to understand how the metabolic costs and benefits from these symbioses with overlapping functions might impact plant growth when evaluating the potential benefits of this engineering strategy. To address this, we developed metabolic models describing how the relative growth rate of *Zea mays* is impacted by the AMF *Rhizophagus irregularis* and a hypothetical N_2_-fixing symbiosis with *Bradyrhizobium diazoefficiens* in isolation and in tandem. The metabolic models of the plant-AMF symbiosis and plant-AMF-rhizobia symbiosis are the first of their kind. To validate the AMF component of our model, we conducted a field evaluation comparing AMF-compatible and mutant AMF-incompatible maize hybrids. The empirically measured AMF-mediated growth benefit agreed well with model predictions. Our model of the rhizobium symbiosis predicted that the lower N content of cereal crops makes the growth penalty associated with acquiring nitrogen from rhizobia smaller than in legumes. Finally, the model of the plant-AMF-rhizobia symbiosis predicted positive synergies between rhizobia and AMF under nutrient-limited conditions but negative synergies under phosphorus-replete conditions. This indicates that these bioengineering strategies could improve cereal crop yields and may achieve greater gains in tandem, but soil nutrient levels and plant nitrogen requirements should be considered.

## Introduction

Agricultural production poses a huge challenge to global efforts to address the intertwined problems of anthropogenic climate change and sustainable agricultural development. Approximately 19% of greenhouse gas emissions can be attributed to agriculture and deforestation (Gates 2021). CO_2_ emissions associated with nitrogen fertilizer production and the release of N_2_O from agricultural fields post-application contribute heavily to these emissions (Boerner 2019; Liu et al. 2020; Jhu and Oldroyd 2023). Additionally, these fertilizers represent a substantial portion of the cost of agriculture, and recent volatility in fertilizer prices has exacerbated food insecurity in Africa (Goodman and O’Reilly 2023). Increases in the cost of urea in Africa specifically have been linked with increased deforestation due to expanded agricultural land use (Kassouri 2024). For these various reasons, researchers are looking to develop alternatives to synthetic fertilizers to allow cultivators, particularly in developing economies, to achieve high crop yields sustainably and profitably.

One promising approach to tackle these challenges is to engineer associations with N_2_-fixing bacteria into crops that do not currently have this capacity (Santi et al. 2013; Guo et al. 2023; Jhu and Oldroyd 2023). If accomplished, this would allow non-leguminous plants, in symbiosis with N_2_-fixing rhizobia, to fix nitrogen instead of relying on external nitrogen applications. This approach could be most effective in a cereal crop with low nitrogen content, like maize (*Zea mays*), since the cost to the plant host to support this symbiosis (ie supplying dicarboxylates to the rhizobia partner to fuel nitrogen fixation) increases with the plant's nitrogen requirements. Due to this lower nitrogen requirement, a previous analysis predicted that maize engineered to host rhizobia would need to provide fewer resources to this symbiosis, compared to legumes like *Glycine max,* when similar levels of N are available, reducing the predicted negative impact on relative growth rate (RGR) (Holland et al. 2023). However, this reduced RGR penalty was inferred through a simple comparison of the nitrogen content of maize and soybean, rather than through a full metabolic modeling analysis. This analysis also did not account for changes in tissue carbon and nitrogen content during maize development, which may result in the growth penalty varying over time. Both of these factors mean that the previous predictions of the RGR impacts of a potential maize-rhizobia symbiosis may be substantial over- or under-estimates. Given the interest in and potential impacts of introducing N_2_-fixation to maize, an updated analysis that can give a more detailed and accurate accounting of these RGR impacts is needed.

In both legumes and non-leguminous crops, these real and potential symbioses with rhizobia exist, or would exist, alongside symbioses with arbuscular mycorrhizal fungi (AMF) (Smith and Read 2010). The plant-AMF symbiosis can confer a variety of benefits to plants, including increased access to soil nitrogen and phosphorus through symbiotic exchange with AMF, which can reduce the amount of fertilizer necessary for optimal crop growth (Beslemes et al. 2023). Because of this, enhancement of this existing capacity for crops to associate with and take up nutrients from AMF has also become a subject of intense research (Hornstein and Sederoff 2024). There is reason to believe that the plant-rhizobia and plant-AMF symbioses may complement each other. The potential growth improvements from N_2_-fixation by rhizobia in cereal crops may be limited in some soils by low phosphorus availability. AMF improves plant phosphorus uptake (Johnson 2010) and could allow plants to fully realize rhizobia-mediated growth benefits in phosphorus-poor soils. A meta-analysis on the effects of AMF-inoculation on plant growth found that in laboratory studies of AMF fungi, N_2_-fixing forbs benefited more from AMF-inoculation than C4 grasses did, suggesting possible synergy between these symbiotic associations (Hoeksema et al. 2010). Positive synergy between rhizobia and AMF has also been reported in white clover (Liu et al. 2023), and another study found that rhizobia also affected the expression of several mycorrhizal genes, including those involved in nutrient transfer to host plants, indicating that partner species can also impact each other's molecular phenotypes (Afkhami and Stinchcombe 2016). Collectively, these data illustrate the diverse molecular mechanisms and transcriptional responses associated with the synergistic benefits of multiple symbionts. These observations make an analysis of this three-species symbiosis and its effects on plant growth highly relevant.

To perform this analysis, models describing (i) the hypothetical symbiosis between a cereal crop and a rhizobium, and (ii) the symbiosis between a cereal crop and AMF, first need to be constructed, before combining them to analyze synergies and antagonisms in the full system. Plant-rhizobia metabolic models have been developed describing legumes, but have not explored the costs and benefits of potential cereal symbioses (Holland et al. 2023), and plant-AMF models have described individual components of the symbiosis (Schnepf and Roose 2006; Schnepf et al. 2016; de Vries et al. 2021), but have stopped short of explicitly connecting plant and fungal metabolism via genome-scale models. In maize specifically, modeling has been done that uses transcriptomic data to predict metabolic alterations in the plant in response to AMF-inoculation, but this is done only with a maize model and without explicitly modeling metabolite exchanges between the plant and AMF (Chowdhury et al. 2023; Decouard et al. 2023). In order to estimate the RGR penalties and exchange stoichiometries of carbon and nutrients between the host plant and AMF, similar to what (Holland et al. 2023) did for the plant-rhizobia symbiosis, a metabolic model connecting the metabolic networks of the plant and AMF is necessary.

To quantify the metabolic costs and benefits of these symbioses, in this manuscript, we present a series of models that explicitly describe the metabolite exchanges between a cereal crop, AMF symbionts, and potential N-fixing rhizobia symbionts both in isolation and in tandem. We then use these models to predict the potential growth benefits and synergies associated with plants’ associations with these symbionts. By interconnecting a maize metabolic model with a genome-scale model of *R. irregularis* (Wendering and Nikoloski 2022), we create the first metabolic model describing the exchange of nutrients between maize and AMF. We generate and present experimental field data comparing hybrid *Zea mays* genotypes that are capable and incapable of associating with AMF and show that our model predictions align with our empirical observations when accounting for available soil nitrogen and phosphorus. Next, we combine our *Zea* mays model with a model of the N_2_-fixing rhizobium *Bradyrhizobium diazoefficiens* (Yang et al. 2017) and use it to calculate the carbon cost of biological nitrogen fixation in this system. Our results confirm previous predictions that the carbon cost should be lower than it is in soybeans, but we also find that this cost would vary across plant development. Using these estimated carbon costs, we perform a cost-benefit analysis that shows that the incorporation of the N_2_-fixing rhizobia into the maize system would be more cost-effective than synthetic N fertilizer applications. Finally, by combining the other models produced in this study, we develop the first models describing potential interactions between the AMF and rhizobia symbioses. These models predict strong synergy between these strategies under low phosphorus and nitrogen conditions in terms of their positive impact on plant RGR (see Figure S1 for a graphical representation of the model). Under phosphorus-replete soil conditions, however, we predict antagonism between these approaches.

## Results

### Construction and validation of a multi-tissue model of maize central metabolism

We constructed a multi-tissue metabolic model of maize central metabolism using a previously published model of *Arabidopsis thaliana*'s central metabolism as a base (Arnold and Nikoloski 2014). This model was adapted to feature a biomass equation representative of maize (Saha et al. 2011), separation of the mesophyll and bundle-sheath cells to simulate C4 photosynthetic fluxes, and separate root and shoot tissue compartments connected by inter-tissue metabolite exchanges based on a previous study (Chowdhury et al. 2023). Where necessary, additional biochemical reactions were added to the model from KEGG and other prior literature sources to allow the synthesis of all biomass components (Dataset S1). Constraints based on empirical measurements are summarized in Table S1.

We compared key outputs of our model, focusing on measures of efficiency and characteristics of C_4_ photosynthesis, to literature estimates and known biochemistry. Model-predicted plant RGRs, photochemical efficiency, and usage of cyclic vs. linear electron flow were highly consistent with the ranges of values reported in the literature (Table 1) (Ehleringer and Björkman 1977; Ermakova et al. 2024; Pace et al. 2024). Key transported and utilized metabolites in C_4_ photosynthesis were also consistent between the model and our current biochemical understanding of this form of photosynthesis (Langdale 2011). Taken together, these validation results indicate that the model captures key photosynthetic details and the overall energetic efficiency of maize central metabolism. A local sensitivity analysis of key parameter values demonstrates that the predicted RGR of our maize model is robust to small variations in these parameters (Figure S2a).

**Table 1:** Photosynthetic and physiological parameter predictions from the model of maize, along with qualitative and quantitative data and corresponding references.

Metric	Modeled	Measured	Reference
Organic Carbon Transport from Mesophyll to Bundle Sheath	Malate	Malate	Langdale (2011)
Carbon Skeleton Transport from Bundle Sheath to Mesophyll	Pyruvate	Pyruvate	Langdale (2011)
Decarboxylated Species in Bundle Sheath	Malate	Malate	Langdale (2011)
Relative Growth Rate (g gDW^−1^ d^−1^)	0.072	0.04–0.08	Pace et al. (2024)
Quantum Yield of Photosynthesis (mol CO_2_ mol photons^−1^)	0.055	0.052; 0.059	Ku and Edwards (1978); Long et al. (1983)
Percent of Bundle Sheath PSI Electrons Supplied by Cyclic Electron Flow (%)	58%	62% ± 22%^a^	Sagun et al. (2019)

^a^Standard error calculated by propagating the errors in the data shown in (Sagun et al. 2019).

### Experimentally validated plant-AMF model relates biomass improvements in maize to nutrient and metabolite exchanges

**Figure 1:**
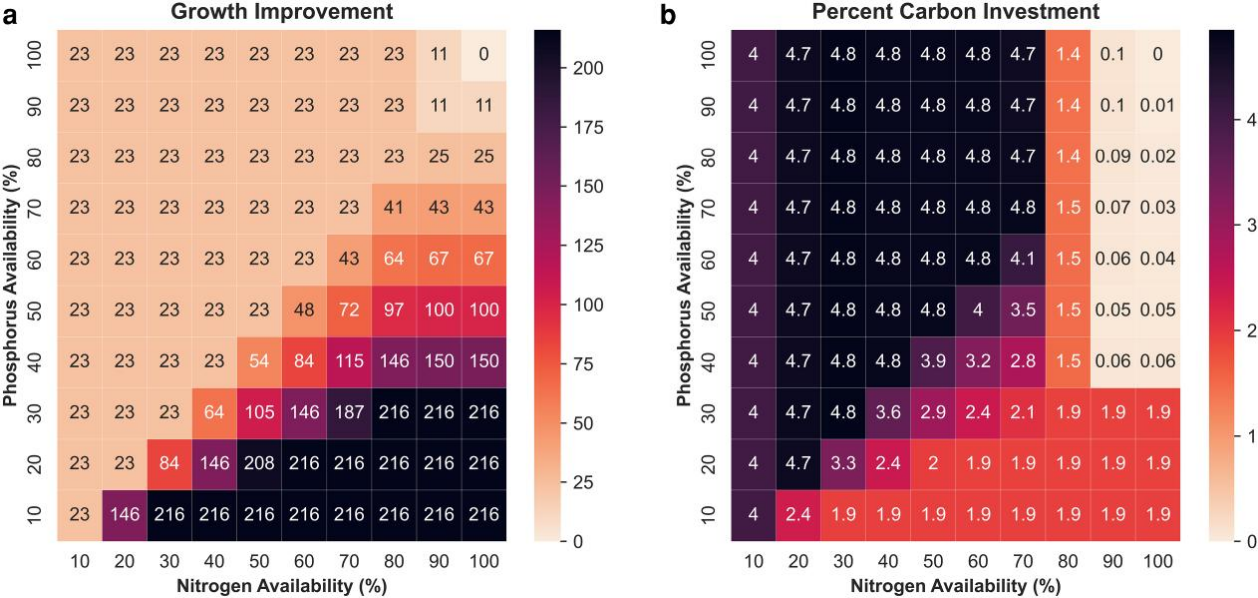
Average growth improvement and carbon investment predictions when comparing with- and without-AMF models when maize biomass is representative of seedling C:N ratios. **(**a**)** Percentage growth improvement of with-AMF vs. without-AMF models of maize as a function of phosphorus and nitrogen availability. **(**b**)** Predicted carbon investment in AMF as a percentage of net CO_2_ assimilation in the plant as a function of phosphorus and nitrogen availability. For plots of growth improvement and carbon investment when the plant biomass is representative of plants at the jointing or silking developmental stages, refer to Figures S4 and S5.

To understand the relationship between the nutrient uptake benefits mediated by AMF and biomass benefits observed in plants, we built a multi-species plant-AMF model by combining our *Zea mays* metabolic model with an existing genome-scale model of the AMF *R. irregularis* (Arnold and Nikoloski 2014; Wendering and Nikoloski 2022). The plant model was allowed to exchange sugars and lipids with the AMF model in exchange for N and P (see Table S1 for key parameters and the Methods section for details of the assumptions underlying the plant-AMF model). Varying the amount of N and P available in the soil for the plant, the model predicted that the presence of AMF has maximal benefit under nitrogen-replete and phosphorus-depleted conditions (Fig. 1a). Accordingly, carbon investment is maximized under the same conditions to support the fungus in exchange for N and P. This carbon investment from the plant decreases, eventually to zero, as nitrogen and phosphorus availability increases and plant uptake alone becomes sufficient to achieve maximum, light-limited plant RGR (Fig. 1b). These predictions are consistent with the trade-balance model of the AMF symbiosis, which describes how the growth benefit conferred by AMF on plants varies due to differing levels of nutrient availability, relative nutrient uptake capacities of plants and AMF, and the biomass composition of plant and fungal tissues (Johnson 2010). By comparing the carbon allocated by the plant to the fungus and the nutrients received in return in our model, we can estimate the stoichiometry of carbon and nutrient exchange between the plant and AMF partner. The rates of these carbon and nutrient exchanges vary with external nutrient levels (Dataset S2). Under strictly P-limiting conditions, the stoichiometry of the carbon-phosphorus exchange is 15 g C g^−1^ P, and under strictly N-limiting conditions, the stoichiometry of the carbon-nitrogen exchange is 7 g C g^−1^ N (Dataset S2).

Our local sensitivity analysis (Figure S2b and c) shows that the stoichiometry predictions from our model vary linearly with the assumed amount of AMF dry weight needed per unit plant dry weight. This means that the accuracy of our estimate of the AMF dry weight needed will determine how reasonable the g C g^−1^ N and g C g^−1^ P estimates are. Using data on the hyphal length density (HLD) of AMF under conditions where phosphorus uptake benefits are observed in plants (Sawers et al. 2017) and literature estimates on the density and fresh-to-dry weight ratio of fungal tissue (Bakken and Olsen 1983), we estimated that AMF biomass amounting to 10% of the total plant biomass is necessary to support maximum nutrient uptake benefits. This is slightly higher but qualitatively similar to a previous study's estimate of ∼6% (Olsson and Johansen 2000). With this assumption, the range of carbon investment predicted by the model, 0 to 5% of plant carbon (Fig. 1b), is consistent with values reported in the literature (2.3 to 10% for *R. irregularis* (Konvalinková et al. 2017)). Due to the relatively small error associated with our estimate of the necessary AMF biomass parameter, its uncertainty does not have an outsized influence on our results or conclusions.

To assess whether the growth-rate improvements predicted by our model are consistent with those observed in the field, we used empirical time-series measurements taken from field-grown maize hybrids with and without a mutation in the gene *Castor* that results in an inability to associate with AMF (Ramirez-Flores 2015). These plants were grown in phosphorus-replete (100%+ of necessary P) and nitrogen-limiting (∼84% of necessary N) soil conditions (Dataset S3). The 95% confidence interval (CI) on the observed RGR benefit when comparing AMF− (n = 12) and AMF + (n = 12) lines in this experimental dataset is 11–47%, which compares well with the predicted 95% CI of 16–19% (no statistically significant difference between distributions is detected at α = 0.05, inferred by overlap of 95% CIs) (Fig. 1a, Dataset S2). This shows that our model accurately predicts AMF-mediated growth benefits under phosphorus-replete, nitrogen-limited conditions. Because our field was N-limited, but not P-limited, to assess the accuracy of our model in P-limited conditions, we compared model outputs to a previous greenhouse experiment conducted under P-limited conditions (Sawers et al. 2017). Under phosphorus-limited conditions, our model predicted a 151–290% improvement in RGR (Fig. 1a), compared to an estimated 66–150% improvement in RGR between AMF + vs. AMF- maize plants (Sawers et al. 2017). This appears to be an overestimate by the model; however, one limitation of our model is that it does not account for changes in biomass composition as a function of nutrient availability and symbiont presence or absence. Although precise biomass composition data is not available, adding demand for phosphorus to the AMF + model corresponding to the 44% increase in biomass P content in the AMF + plants in (Sawers et al. 2017), we predict a 72–167% improvement, consistent with what was measured. In the case of the comparison with the N-limited field data presented in the present study, AMF + plants have 2.5 times the P content of the AMF- plants (Dataset S3). Revising these predictions to account for the greater P content of the AMF + plants result in a predicted improvement 95% CI of 16–19%. This is unchanged from the original prediction because P is non-limiting in this case. We have not made a similar correction to account for changes in N content between AMF- and AMF + plants because this data is unavailable for the comparison with (Sawers et al. 2017) and there is no statistically significant difference in the proportion of tissue biomass made up of N between AMF + and AMF- plants in the field data according to a two-sided Student's T-test (α = 0.05; n = 38).

Since (Sawers et al. 2017) do not report time-series dry weight measurements, we estimated the experimental RGR using the final dry weight measurements after 8 wk compared to a typical kernel weight of 2.5 g. This introduces uncertainty in the RGR improvements inferred from (Sawers et al. 2017) that cannot be readily quantified. As such, there is an added layer of uncertainty in the experimental RGRs calculated from this greenhouse study that is not present in the RGRs calculated from the N-limiting field experiments

### The predicted RGR penalty incurred by N_2_-fixation in maize is lower than in non-cereal crops and decreases across vegetative development

**Figure 2:**
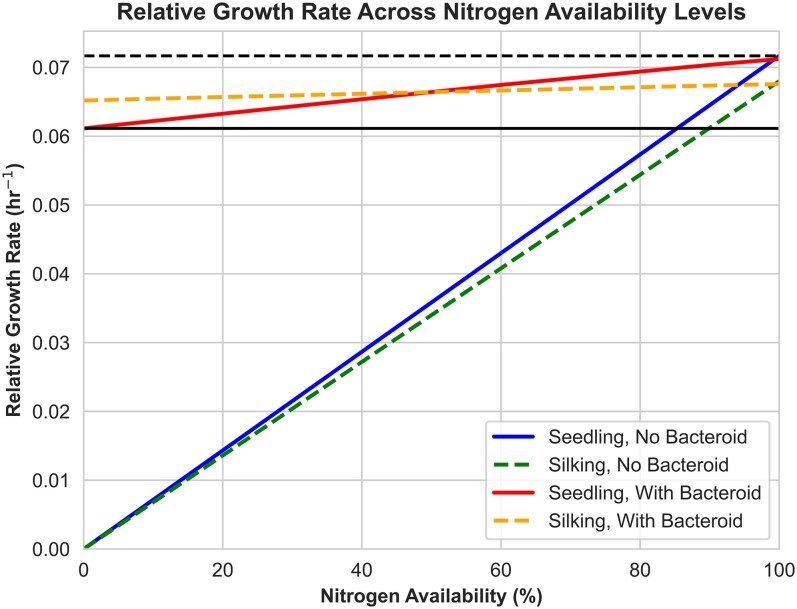
Relative growth rate of *Z. mays* across a range of nitrogen availability levels for models with biomass representative of maize seedlings and maize plants at silking. Solid lines represent growth predictions when using the seedling biomass equation, and dashed lines represent growth when using the silking biomass equation. The dashed black line represents the maximum growth rate of the non-nodulated model when it receives all its nitrogen from the soil. The solid black line represents the maximum growth rate of the nodulated model when it fully relies on N_2_-fixation. For the relative growth rates of the models with biomass representative of the C:N ratios of maize plants at the jointing stage, see Figure S6.

In order to investigate the potential benefits and drawbacks of creating an association between maize and an N_2_-fixing rhizobium, a model of *B. diazoefficiens* (Yang et al. 2017) was connected to the maize model, with a new nodule tissue compartment. By comparing simulation results with and without the rhizobia and related plant tissues present, we quantified the RGR penalties of using N_2_-fixation in lieu of soil nitrogen uptake across a range of soil nitrogen availabilities and across several different vegetative developmental stages in maize (Table 2; Fig. 2).

**Table 2:** Physiological and metabolic predictions from the *Z. mays* model associated with the N2-fixing rhizobium *B. diazoefficiens.*

Metric	Timepoint/Percent n of biomass (%)		
	Seedling/2.70	Jointing/2.34	Silking/0.80
Relative Growth Rate (g gDW^−1^ d^−1^)	0.071	0.07	0.068
Relative Growth Rate Penalty (%)	14.7	12.9	4.1
N_2_-Fixation (mmol gDW^−1^ d^−1^)	0.059	0.052	0.019
Nodule CO_2_ Efflux (mmol gDW^−1^ d^−1^)	0.37	0.33	0.14
Carbon cost of N_2_-Fixation (g C g^−1^ N)	2.7	2.7	3.3

The model predicts that rhizobial symbiosis exerts an early small cost to growth (∼14.7% at the seedling stage) that decreases over the course of plant development (4.1% at the silking stage). These numbers are qualitatively consistent with prior estimates (Holland et al. 2023). The predicted carbon cost of N_2_-fixation ranges from 2.7 g C g^−1^ N at the seedling stage to 3.3 g C g^−1^ N at the silking stage. This decreased efficiency results from fixed non-growth-associated maintenance in the nodule and bacteroid tissues with decreased use of N_2_-fixation during the silking stage when nitrogen requirements begin to decrease (Table 2). These values are consistent with the range of empirically measured g C g^−1^ N values in the literature for soybean (2.5 to 7 g C g^−1^ N) (Warembourg 1983). In summary, our model results suggest that *Z. mays* could be a promising host for a novel N-fixing rhizobia symbiosis because its low N content could allow it to rely on biological N-fixation with much smaller growth penalties than have been predicted in legumes.

### Value cost ratio comparisons indicate that a nitrogen-fixing maize crop could be profitable in both the United States and sub-Saharan Africa

To assess the economic viability of inoculating a hypothetical nodulating maize plant with the modeled RGR penalties, we calculated the Value Cost Ratio (VCR) of fertilizing maize with typical nitrogen fertilizer and the VCR of adding rhizobia inoculum with yield and price estimates for the United States (USA) and sub-Saharan Africa (SSA) (Table 3). The VCR represents the ratio of the economic benefit associated with a given intervention (eg application of fertilizer) to the cost of implementing that intervention. A value of 1 represents the break-even point between costs and benefits, and values greater than 1 represent positive returns on investment.

**Table 3:** VCR and input parameter values comparing the profitability of fertilizer and rhizobia inoculum application versus using no fertilizer or inoculum amendments in the USA and SSA.

Source	Parameter	Value	Condition	VCR
USA	Fertilizer price (USD ha^−1^)	198 (Schnitkey et al. 2022)	+ Fertilizer	3.4
	Yield with fertilizer (tonne ha^−1^)	11.5 (United States Department of Agriculture: $6#?>National Agricultural Statistics Service 2024a)		
	Inoculum price (USD ha^−1^)	9.9 (Links et al.)	+ Inoculum	44.3
	Yield with inoculum (tonne ha^−1^)	9.8 (United States Department of Agriculture: $6#?>National Agricultural Statistics Service 2024a)^a^		
	Corn price (USD tonne^−1^)	141 (United States Department of Agriculture: $6#?>National Agricultural Statistics Service 2024b)	Control	
	Control Yield (tonne ha^−1^)	6.7 (Bayer Crop Science 2024)^b^		
SSA	Fertilizer price (USD ha^−1^)	232 (Bonilla-Cedrez et al. 2021)^c^	+ Fertilizer	2.3
	Yield with fertilizer (tonne ha^−1^)	3.6 (Bonilla-Cedrez et al. 2021)		
	Inoculum price (USD ha^−1^)	38 (Ulzen 2018; Holland et al. 2023)	+ Inoculum	10.7
	Yield with inoculum (tonne ha^−1^)	3.1 (Bonilla-Cedrez et al. 2021)^a^		
	Corn price (USD tonne^−1^)	230 (Bonilla-Cédez)	Control	
	Control Yield (tonne ha^−1^)	1.3 (Bonilla-Cedrez et al. 2021)		

^a^Yield with inoculum calculated by assuming that relative growth rate penalties translate 1:1 into yield penalties. We assume the worst-case scenario for the use of nitrogen-fixing maize by applying the largest relative growth rate penalty observed (14.7% penalty at the seedling stage).

^b^Control yield value taken from a report on a plot that had not had fertilizer amended for 2 yr (Bayer Crop Science 2024).

^c^Taken from average fertilizer application needed to achieve optimal yield across a range of SSA sites in the mechanistic and empirical models presented in Bonilla-Cedrez 2021 (Bonilla-Cedrez et al. 2021).

Our results show that in both the USA and SSA, the potential profitability of a nitrogen-fixing maize crop is greater than that typically observed with regular nitrogen fertilizer additions, with a VCR of 44.3 vs 3.4 for the USA and a VCR of 10.7 vs 2.3 for SSA, respectively. The greater potential profitability in the USA is driven by the much lower price of inoculum in the USA ($9.9 ha^−1^)(Links et al.) as compared with SSA ($38 ha^−1^)(Ulzen 2018; Holland et al. 2023). This large difference in inoculum cost contrasts with the similar nitrogen fertilizer costs in the USA ($198 ha^−1^)(Schnitkey et al. 2022) and SSA ($205 ha^−1^)(Bonilla-Cedrez et al. 2021). Relying on N-fixation in a hypothetical maize-rhizobia system could be ∼5–13 times more economical than synthetic fertilizer application, with inoculum cost representing the biggest variable in determining just how effective this strategy would be.

### The presence of AMF is predicted to stabilize RGR benefits in maize that is in symbiosis with N_2_-fixing rhizobia

**Figure 3:**
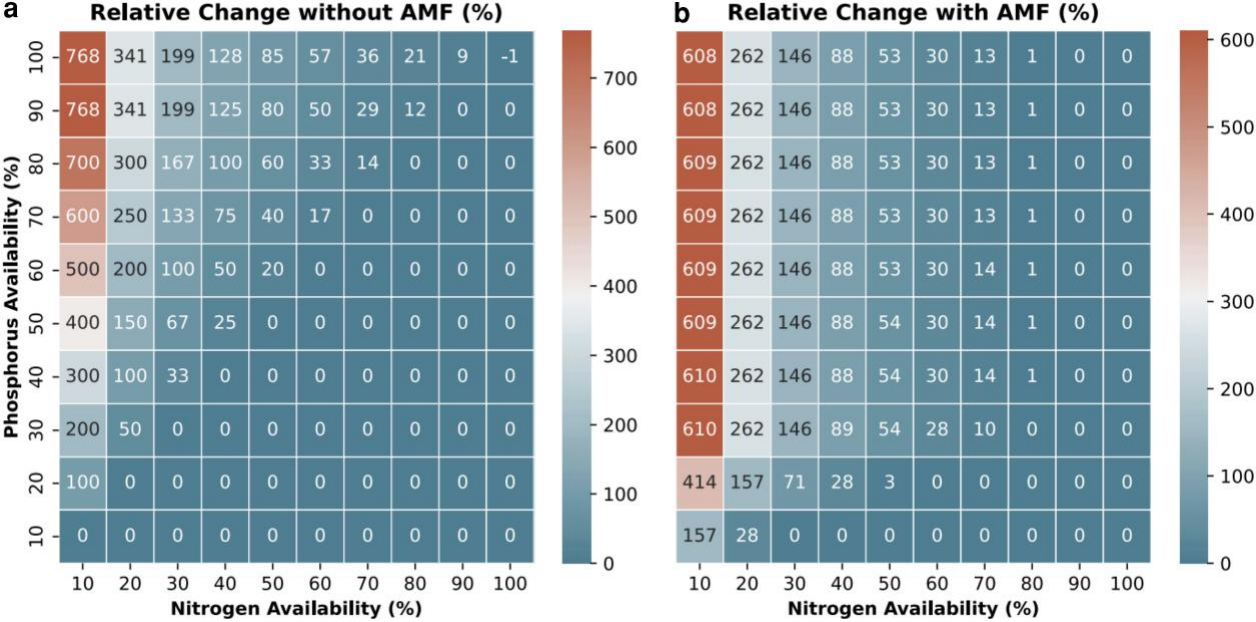
Comparison of predicted RGR improvement resulting from the addition of N_2_-fixing rhizobia to *Z. mays* when AMF is absent **(**a**)** and present (b). Relative change in both cases is calculated by taking the difference in RGR between a base case and that same model with the rhizobia added—in **(**a**)** this is the difference between the *Z. mays* model alone and the *Z. mays* + *B. diazoefficiens* model, and in **(**b**)** this is the difference between the *Z. mays* + *R. irregularis* model and the *Z. mays* + *B. diazoefficiens* + *R. irregularis* model.

To quantify the impact *Zea mays’* existing AMF symbioses may have on the efficacy of engineering a novel symbiosis with an N_2_-fixing rhizobium, we constructed a model featuring maize in association with both symbionts and then compared the predicted RGR improvements from adding rhizobia to maize models with and without AMF (Fig. 3).

When AMF is not present, the addition of rhizobia results in improved plant RGR, with benefits being most pronounced when external nitrogen is low (Fig. 3a). However, in order to realize these RGR benefits, sufficient external phosphorus is needed to support additional biomass accumulation from rhizobia-provided nitrogen. The dependency of rhizobia-mediated RGR benefits on phosphorus is greatly reduced when the AMF symbiosis is present (Fig. 3b), due to the dramatic P uptake benefits conferred by the AMF. This leads to more consistent gains in RGR across the profile of external N and P availability, with the differences most pronounced under extremely N-deprived and moderately P-deprived conditions. This observation indicates that there can be synergy between the rhizobia- and AMF-mediated growth benefits under at least some soil conditions.

### Rhizobia and AMF provide synergistic benefits under nutrient-poor conditions, but are antagonistic under nutrient-replete conditions

To better understand the conditions under which rhizobia and AMF-mediated nutrient benefits complement or conflict with one another, we predicted growth benefits across different phosphorus and nitrogen levels with the full maize-rhizobia-AMF model and compared them with the additive benefits of using each strategy in isolation. By calculating the difference between the RGR benefits predicted by the combined model and the additive predicted benefits from the individual plant-symbiote models (Figure S3), we were able to quantify the synergy and antagonism between these strategies as a function of nutrient availability.

**Figure 4:**
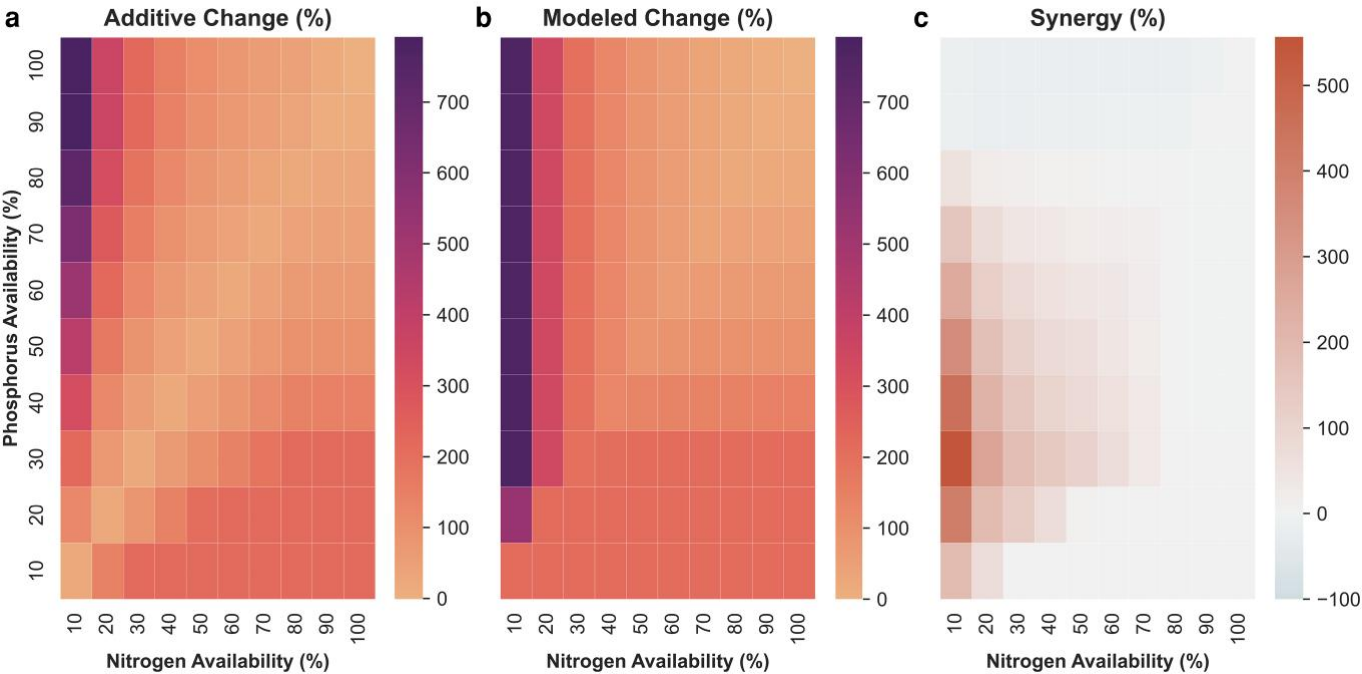
Additive and modeled RGR benefits and calculated synergies and antagonisms in the *Zea mays*, *Bradyrhizobium diazoefficiens*, *Rhizophagus irregularis* three-species system when the maize model's biomass is representative of seedling C:N ratios. **(**a**)** The sum of relative growth rate changes from adding *B. diazoefficiens* or *R. irregularis* to the *Z.* mays model under given nitrogen and phosphorus availabilities. **(**b**)** Modeled relative growth rate changes in the three-species model. **(**c**)** Synergy and antagonism between *B. diazoefficiens* and *R. irregularis* were quantified as the difference between the modeled and additive growth rate changes. For the additive change, modeled change, and synergy when the maize model's biomass is representative of plants at the joining or silking stages, refer to Figures S7 and S8.

Under phosphorus depletion and low to medium nitrogen availability, our model predicts that the 2 symbiotic relationships work synergistically by relaxing the nutrient limitation imposed by both phosphorus and nitrogen. This synergy allows the plant to achieve near-optimal RGR with less than 100% N and P availability. Maximal RGR improvement is observed under conditions of phosphorus depletion with low to moderate nitrogen availability (Fig. 4b). In this new maximal region, the relative benefit increased from 23% using only AMF or 200% using only rhizobia to 779%, corresponding with an absolute increase of RGR by 0.056 d^−1^. The synergism between the symbiotic relationships is largest (556%) when nitrogen availability is at 10% and phosphorus levels are at 30% (Fig. 4c). The predicted RGR of plants grown under these nutrient conditions with both the AMF and rhizobia symbiosis is 0.061 d^−1^, almost triple the value predicted assuming the rhizobia and AMF are non-synergistic (0.024 d^−1^). Comparing this with the RGR predicted for plants without either symbiont but with 100% of their nutrient needs met (0.072 d^−1^) suggests that plants could achieve ∼85% of their maximum RGR with only 10% and 30% of optimal N and P availability. Under these conditions, the maize plants would get the majority of their N from their rhizobial symbionts (∼86%) and only (∼3%) from their AMF symbionts **(**Dataset S2**)**, consistent with the lower g C g^−1^ N associated with rhizobia versus AMF. The synergy in these conditions appears to be driven by the AMF alleviating the P-limitation on growth that the plants experience when only the rhizobia are present.

Maximum antagonism (−20%) is observed under phosphorus-replete conditions, whereas the model predicts negligible antagonism (less than −1%) under nitrogen-replete conditions. The magnitude of the positive synergy under moderate- to low-nitrogen availability and moderate phosphorus availability, expressed in terms of relative change (48–556%), far outweighs the worst antagonisms seen (0–20%). However, the worst antagonisms are observed under phosphorus-replete conditions, some of which support high basal RGR. This causes the worst antagonism in terms of absolute RGR differences under phosphorus-replete conditions (−0.014 d^−1^) to be closer to the highest positive synergy values (0.035 d^−1^) than one would expect based purely on the relative changes.

## Discussion

In this study, we present the first metabolic model describing the interconnected metabolism and nutrient exchanges between a plant and an AMF. We provide experimental validation of the predicted relationship between these exchanges and plant growth benefits. We also built on previous analyses performed in (Holland et al. 2023) by building the first metabolic model of a hypothetical association between a cereal crop and monocot, *Z. mays*, and an N-fixing rhizobium. Analysis of this model supported the conclusion in (Holland et al. 2023) that cereal crops could rely on biological nitrogen fixation with a smaller penalty to their growth rate than what we expect to see in agricultural legumes, while also showing that the predicted growth rate penalties vary depending on the developmental stage of the plant. A simple economic analysis showed that, if successfully engineered, inoculation and cultivation of a maize-rhizobium system could outcompete the use of synthetic fertilizers from a cost-benefit perspective, but that this strategy would currently be more attractive in the United States than in a developing region like sub-Saharan Africa due to differences in inoculum prices. Finally, we created a combined plant-AMF-rhizobia model, which predicted substantial synergy between the 2 symbioses when nitrogen and phosphorus availability is low and antagonism when phosphorus availability is adequate. Our results indicate that these engineering strategies could efficiently and economically improve cereal crop yields, but that local soil nutrient conditions should be considered in order to maximize potential growth benefits.

### Modeling the benefits of AMF on plant nutrition and growth

To our knowledge, this study presents the first metabolic model describing the exchange of carbon, nitrogen, and phosphorus between a plant and AMF. Our plant-AMF framework reproduced empirically observed RGR benefits and predicted carbon investments consistent with prior literature (Konvalinková et al. 2017) and represents a promising platform for computational investigations into plant-AMF symbiosis. This framework could be further strengthened with additional information on below-ground AMF biomass quantities and the interdependence of nitrogen uptake, phosphorus uptake, and plant biomass gains. The data currently available is limited and has widely varying estimates of the impacts of AMF, particularly regarding AMF-mediated nitrogen uptake (Reynolds et al. 2005; Wang et al. 2018; Beslemes et al. 2023).

### Impact of existing AMF symbiosis on potential benefits of rhizobia engineered into maize

Considering the potential benefits of adding rhizobia to cereal crops, it is crucial to understand how possible interactions of this engineered symbiosis with the existing AMF associations complement or hinder each other. Our model predicts that a maize plant with this symbiosis could achieve substantial and consistent growth benefits across a wider range of soil nutrient conditions than a plant without this AMF symbiosis. We predict that synergy between the AMF and rhizobia strategies roughly doubles the RGR of maize plants under severe N deprivation and moderate P availability compared to a scenario where no synergy between these strategies exists. We also predict significant antagonism between these strategies under phosphorus-replete conditions. These findings suggest that existing AMF symbioses are likely to enhance the benefits of a hypothetical rhizobia symbiosis in cereal crops, but also that redundancy between the symbionts under phosphorus-replete conditions should be considered. These findings also provide a potential explanation for reported synergies between rhizobia and AMF and the observation that the growth of plants hosting N_2_-fixing rhizobia seems to benefit more from AMF inoculation than C4 grasses that lack the symbiosis (Hoeksema et al. 2010; Liu et al. 2023). According to our model, these positive synergies may be attributed to AMF allowing plants associated with rhizobia to overcome P limitation.

### Potential growth impacts of engineering N_2_-fixing rhizobia symbiosis into maize

Both the addition of rhizobia to cereal crops and the enhancement of AMF-associations and accompanying nutrient-uptake benefits are strategies being pursued to sustainably improve agricultural production (Guo et al. 2023; Jhu and Oldroyd 2023; Hornstein and Sederoff 2024), so the models presented in this study describing the maize-rhizobia and maize-AMF symbioses in isolation are also important to consider. We explored the efficiency of engineering rhizobia N_2_-fixation and enhanced AMF association individually. We predict a range of RGR penalty values that includes, but is generally lower than the ∼14% predicted in previous work (Holland et al. 2023). We also find that the RGR penalty is highest earlier in vegetative growth when the nitrogen content of plant tissue is higher, so focusing on end-point measurements of plant biomass composition may not give the best estimate of the cost of nitrogen fixation over a growing season. However, our results support the idea that cereal crops should experience smaller RGR losses than legumes when relying on N_2_-fixation (Table 2), making this an attractive bioengineering approach to sustainably improve cereal crop yields.

### Economic viability of engineering N_2_-fixing rhizobia symbiosis in maize

Our VCR analysis (Table 3) indicates that inoculation of a nitrogen-fixing maize plant could be a profitable alternative to the use of nitrogen fertilizer in both the USA and SSA. However, the profitability would be greater in the USA due to the almost fourfold greater price of inoculum on a per-hectare basis in SSA as compared with the USA (Bonilla-Cedrez et al. 2021; Holland et al. 2023). This suggests that, if nitrogen-fixing maize is developed, reducing the cost of inoculum for cultivators will be important for driving the adoption of this technology in SSA. One caveat to this analysis is the great deal of variability in maize yield potential seen across SSA, with some regions having much higher potential yields upon fertilization, closer to 7 tonne ha^−1^ as compared with the 3.6 tonne ha^−1^ used in our analysis (Bonilla-Cedrez et al. 2021). Another caveat is that the degree to which inoculation with rhizobia is necessary to achieve good levels of biological nitrogen fixation varies with the natural abundance of the particular species. Here, we have modeled *Zea mays* in a hypothetical association with *B. diazoefficiens*, but other species could require less inoculum per hectare, making the cost of inoculum less influential in the cost-benefit analysis. Finally, other factors in the field, such as competition from other members of the soil microbiome, and the impact of local environmental conditions on the effectiveness of inoculation and the ability of microbes to associate with and confer benefits on plant hosts, are not factored into our model or the VCR analysis.

### Limitations and future directions

A major limitation of the models presented in this study is the fact that only metabolic interactions are considered. While a model incorporating all of the diverse regulatory details and potential interactions between plants, rhizobia, and AMF would not be possible given our current knowledge of these systems, it should be noted that unmodeled factors, such as water-use impacts or regulatory cross-talk between rhizobia and AMF, may greatly impact the real-world system. This prevents us from using these models to ask questions about the costs and benefits of these symbioses under some agronomically relevant scenarios, such as drought conditions. Additionally, there is room to expand on the models presented in this study to further investigate the costs and benefits of symbiosis on maize.

Although we have made an effort to partially explore the impact of the plant's developmental stage on the costs and benefits of the rhizobia symbiosis, a quantitative model describing how symbiont presence/absence and soil nutrient levels affect biomass composition would greatly enhance the predictive capabilities of the presented metabolic models. The *R. irregularis* model could be expanded to account for the different tissue types seen in that organism (Wendering and Nikoloski 2022). As described in the Methods, the *Z. mays* model used in this study was adapted from an *A. thaliana* base model and focuses on core metabolism. As such, any effects resulting from the specialized metabolism of *Z. mays* on growth are not captured. The present model, therefore, would be ill-suited, for example, to describe the costs associated with the production of signaling molecules necessary for the establishment of plant-AMF and plant-rhizobia symbioses. However, such factors could be modeled in future studies using more expansive genome-scale models of *Z. mays*. The present model also uses static optimization of a small number of discrete time points, instead of attempting to model the plant's growth over the course of a growing season. Because of this, the model cannot predict how the RGR penalties at one growth stage might affect the overall growth trajectory of a plant. Adoption of a dynamic FBA (Mahadevan et al. 2002) approach or integration with a crop growth model, as in (Holland et al. 2023), could be used to address this.

Further, in our framework, the fungus is treated as carbon-limited and does not compete for external nutrients with the plant, so the parasitism predicted by the trade-balance model (Johnson 2010) under nutrient-replete conditions is not predicted by our model. We therefore expect that our model's predictions will underestimate the growth rate penalty incurred by the AMF symbiosis when soil nutrient levels are abundant. Explicitly modeling this resource competition could address this. Finally, to quantify the impact of these symbioses on grain production, a developing kernel tissue compartment could be added to the maize model. Incorporating the predicted dynamic changes to RGR corresponding to the different developmental stages with a crop growth model would then allow us to translate RGR changes into yield predictions. In light of recent interest in developing maize lines with modified kernel N-content and a growing season optimized to better make use of soil N, a modeling framework that integrates detailed flux maps with yield potential in the context of symbioses that affect plant nutrition could be very valuable (Ojeda-Rivera et al. 2025). While currently beyond the scope of our model, this model provides a foundation that could be further expanded to provide insight into these questions.

## Materials and methods

This study involved the assembly of 4 distinct metabolic models:

1. A shoot/root model of *Z.* mays central metabolism with differentiated bundle sheath and mesophyll cell metabolism.
2. Model (1) interconnected with the AMF *R. irregularis*.
3. Model (1) interconnected with the rhizobia *B. diazoefficiens*.
4. Model (1) interconnected with both the AMF *R. irregularis* and the rhizobia *B. diazoefficiens*.

Descriptions of decisions made in constructing these models can be found below, along with key literature-derived constraint values (summarized in Table S1). Version-controlled model code and analysis scripts necessary to reproduce the simulations performed in this study can be found at https://github.com/Matthews-Research-Group/Maize-Rhizobia-AMF_FBA. All simulation results reported in this study were produced by running the code in commit b8ca111 in the GitHub repository.

### Maize shoot/root model construction

For this study, a reconstruction of *Zea mays* metabolism was needed that (i) represents the separation of the mesophyll and bundle sheath cells to accurately represent C4-associated metabolic costs and (ii) is computationally tractable in terms of model size. While several generic C4 and *Zea mays* specific reconstructions exist (de Oliveira Dal’Molin et al. 2010a; Saha et al. 2011; Seaver et al. 2015; Chowdhury et al. 2023), none of them addressed both of these needs. We constructed a multi-tissue metabolic model of maize central metabolism using a bottom-up reconstruction approach using the central metabolism of *Arabidopsis thaliana* as a base (Arnold and Nikoloski 2014). This model was chosen for its extensively vetted description of core metabolism, relatively small size, and previous use in developing models of C_4_ metabolism (Blätke and Bräutigam 2019). As done in a previous C_4_ metabolic modeling study (Blätke and Bräutigam 2019), this model was duplicated to create separate bundle sheath (BS) and mesophyll (M) models. Other additions and refinements of the bottom-up reconstruction (Arnold and Nikoloski 2014) described in the C_4_ modeling study (Blätke and Bräutigam 2019) were also made to improve the accuracy of the model, with the exception of the latter's modifications to the chloroplast ATP synthase reaction, since the original model already correctly captured the c-subunit stoichiometry and consequent ATP/proton stoichiometry typical of plant systems (Hahn et al. 2018).

These models were then connected by transport reactions representing exchanges between BS and M cells through the plasmodesmata, and flux through cytosolic phosphoenolpyruvate carboxylase and mitochondrial malate dehydrogenase 1 were disallowed, based on the methods described in (Blätke and Bräutigam 2019), to ensure that the flux map of the resulting model lines up with canonical carbon assimilatory fluxes through the NADP-ME subtype characteristic of maize (Hatch 1987). As in the earlier soybean-rhizobium FBA study (Holland et al. 2023), and based on earlier literature derivations (Farquhar et al. 1980), an upper bound of 2,920 µmol g^−1^ DW h^−1^ on photon uptake was imposed.

Another duplicate of the central metabolic model (Arnold and Nikoloski 2014) was then made to represent the plant's root tissue. Metabolite exchanges were adopted from (Chowdhury et al. 2023). All metabolite exchanges between the shoot tissue model and the environment, apart from CO_2_, O_2_, and light, were disallowed. The root tissue model was treated the same, except with the additional capacity to take up ammonium, orthophosphate, water, and dihydrogen sulfide. The orthophosphate uptake reaction was modified based on the fact that direct phosphate uptake is thought to occur through proton symporters (Chiu and Paszkowski 2019). Two protons were assumed to move across the cell membrane for each phosphate (Dreyer et al. 2019).

Base biomass compositions for the root and shoot tissue were adapted from (Saha et al. 2011), with additional reactions from KEGG (Kanehisa 2002) added to produce biomass components not covered by the original *Arabidopsis* central metabolism model (Arnold and Nikoloski 2014). A list of added reactions can be found in Dataset S1. The ratio of shoot to root biomass was constrained to a value of 90:10 (Su et al. 2019).

Growth-associated maintenance costs (GAM) and non-growth-associated maintenance costs (NGAM) for maize were calculated from literature data (De Vries et al. 1979) and modeled either as generic ATP or glucose burning at a specified rate **(**Dataset S4**)**. Growth-associated maintenance was calculated as 19 mmol ATP gDW^−1^ d^−1^, lower than the assumed 30 ATP gDW^−1^ d^−1^ featured in many other plant metabolic models (de Oliveira Dal’Molin et al. 2010b). The NGAM cost was calculated as 5.76 mmol ATP gDW^−1^ d^−1^. All maintenance costs were distributed across tissues according to their proportions of total dry weight.

### AMF maize model construction and analysis

We used the previously published and validated GEM of the AMF *R. irregularis* to represent the fungal partner in a plant-fungus symbiotic relationship (Wendering and Nikoloski 2022). To make the *R. irregularis* model dependent on the plant for its carbon and energy needs, all external uptakes were disabled except for those with oxygen, carbon dioxide, dihydrogen sulfide, ammonium, phosphate, and a number of mineral micronutrients necessary for biomass accumulation. Based on literature evidence, only a small number of metabolites were allowed to be exchanged between the fungal and plant root models (Chiu and Paszkowski 2019) (Dataset S5). Prior literature suggests that plants allocate carbon to AMF symbionts in the form of hexoses, primarily glucose (Bago et al. 2000; Wipf et al. 2019), and lipids, most likely in the form of palmitate (Olsson and Johansen 2000; Luginbuehl et al. 2017). In our plant-AMF model, these carbon sources are exchanged for nitrogen (represented as NH_4_ in our model) and phosphorus taken up by the AMF. From the plant, sugar (in the form of glucose) and palmitic acid were allowed to be transported to the AMF. From the fungus, phosphate and ammonium were allowed to be transported, with a cost of 0.5 ATP per orthophosphate transported (Dreyer et al. 2019).

### Recalculation of biomass coefficients to account for changing tissue C:N ratios

Published C:N ratio data (Li et al. 2022) for *Zea mays* was used by modifying the base maize biomass composition from Saha et al. (Saha et al. 2011) to match the measured shoot and root C:N ratios. The nitrogenous biomass (NB) and non-nitrogenous components of the biomass (B) were grouped, and the appropriate proportion for these groups was calculated as follows:


(1) $$\begin{array}{c} NB_{\mathrm{proportion}}=\frac{B_{\mathrm{carbon}\;\mathrm{proportion}}}{C:N\times NB_{\mathrm{nitrogen}\;\mathrm{proportion}}-NB_{\mathrm{carbon}\;\mathrm{proportion}}-B_{\mathrm{carbon}\;\mathrm{proportion}}} \end{array}$$

where *B*_carbon proportion_ is the proportion of the weight of the non-nitrogenous biomass that is made up of carbon, *NB*_nitrogen proportion_ and *NB*_carbon proportion_ are the proportions of the weight of the NB that is made up of nitrogen and carbon, respectively, and *C:N* is the desired C:N ratio. We then split the proportion of non-nitrogenous biomass into the individual non-nitrogenous biomass components in a way that maintains their ratios relative to each other from the original biomass equation (Saha et al. 2011). Finally, these weight proportions were divided by the average molecular weight of each class of compounds to give the coefficients for each in the final biomass equation in units of mmol gDW^−1^ d^−1^.


(2) $$\begin{array}{c} \mathrm{Nitrogenous}\;\mathrm{compound}s_{\mathrm{coefficient}}=\left(\frac{1000\mathrm{mmol}}{\mathrm{mol}}\right)\left(\frac{NB_{\mathrm{proportion}}}{MW_{\mathrm{Nitrogenous}\;\mathrm{compounds}}}\right) \end{array}$$

(3) $$\begin{array}{rl} & \mathrm{Coefficien}t_{n}=\left(\frac{1000\mathrm{mmol}}{\mathrm{mol}}\right)\\ & \;\;\;\times \left(\frac{(1-NB_{\mathrm{proportion}})\left(\frac{\mathrm{Original}\;\mathrm{Coefficien}t_{i}}{\sum_{i\in \mathrm{Biomass}\;\mathrm{Coefficients}}\mathrm{Original}\;\mathrm{Coefficien}t_{i}}\right)}{\mathrm{Average}\;MW_{i}}\right) \end{array}$$

where Original Coefficient*_i_* is the coefficient for a given biomass component *i* in the original biomass equation (Saha et al. 2011), Biomass Coefficients is the set of all of these original coefficients, and *Average MW_i_* is the average molecular weight of the constituent metabolites in a group of biomass compounds. Note that the final coefficients from this calculation result in biomass equations whose components add up to 1 gDW.

### Nodulated maize model construction and analysis

The workflow previously used in (Holland et al. 2023) was adapted to create a multi-species model with the maize C4 root/shoot model described above as the plant component and the GEM of *B. diazoefficiens* as the N_2_-fixing bacterial symbiont (Yang et al. 2017). This workflow was used because it was shown to produce reasonable growth and N-fixation efficiency predictions, as well as to allow for ease of comparison between the results in the present study and prior work.

First, a duplicate of the root model, representing the tissue of a hypothetical maize nodule, was made with exchanges between it and the root limited as in Holland et al. (Holland et al. 2023) and as listed in Dataset S5. Next, transporters describing the exchange of dicarboxylates and ammonium were added between the nodule model and the *B. diazoefficiens* model to reflect known exchanges from the literature (Holland et al. 2023). Allantoate is thought to be the primary form of nitrogen exchanged between nodule tissue and the plant root, so reactions describing allantoate biosynthesis and degradation were added to the model from KEGG (Kanehisa 2002) (Dataset S1).

Because *B. diazoefficiens* specific values were unavailable, the non-growth associated maintenance (NGAM) costs of *B. diazoefficiens* were assumed to be the same as those for the *Medicago truncatula* bacteroid *Sinorhizobium meliloti*: 2.52 mmol gDW^−1^ h^−1^ (diCenzo et al. 2020). Assuming that the bacteroid constitutes 2% of the biomass of the overall system, as in (Holland et al. 2023), this value was scaled to 1.21 mmol gDW^−1^ d^−1^. At steady-state we assume that the bacteroid is not accumulating biomass, so growth-associated maintenance costs were ignored. Note that the *M. truncatula/S. meliloti* symbiosis features indeterminate nodule formation and amide export, in contrast to the determinate nodules and ureide export of the *Glycine max/B. diazoefficiens* symbiosis we are elsewhere modeling the hypothetical *Z. mays*/*B. diazoefficiens* symbiosis after. These differences likely result in differences in NGAM; however, as shown in Figure S2, model outputs are relatively robust to changes in NGAM and associated parameters, like tissue proportions, suggesting that small variations in these costs will not have a large impact on study conclusions.

### Optimization details

Flux Balance Analysis optimizations were performed, in most simulations, to maximize plant RGR.


(4) $$\begin{array}{c} \max v_{\mathrm{plant}\;\mathrm{biomass}} \end{array}$$

s.t.

$$\begin{array}{c} Sv=0 \end{array}$$

$$\begin{array}{c} LB_{j}\leq v_{j}\leq UB_{j} \end{array}$$

where *S* represents the stoichiometric matrix of the metabolic network model being optimized, *v* is the vector of all fluxes in that network, and *LB* and *UB* are vectors of the lower and upper bound constraints. In this study, all optimizations were followed up by minimization of the sum of fluxes in the network via regularization of the L1-norm (Holzhütter 2004), often referred to as parsimonious FBA (pFBA) (Lewis et al. 2010), which corresponds to the following optimization:


(5) $$\begin{array}{c} \min\underset{\;j\in \mathrm{Reactions}}{\sum }\;v_{j} \end{array}$$

s.t.

$$\begin{array}{c} Sv=0 \end{array}$$

$$\begin{array}{c} LB_{j}\leq v_{j}\leq UB_{j} \end{array}$$

$$\begin{array}{c} v_{\mathrm{plant}\;\mathrm{biomass}}=v_{\mathrm{maximized}\;\mathrm{plant}\;\mathrm{biomass}} \end{array}$$

where *j* is the index of a reaction in the list of all reactions in the network (*Reactions*) and *v*_maximized plant biomass_ is the maximized biomass value obtained from the initial optimization.

### Model optimization and analysis

Growth benefits associated with N_2_-fixation, AMF association, and both N_2_-fixation and AMF association in concert, were estimated by comparison of predicted plant RGR in the base maize model and the appropriate symbiosis models across a range of soil nitrogen and phosphorus uptake rates (Figure S2). All nitrogen uptake by the plant and, in models that feature it, the AMF symbiont is assumed to be in the form of ammonium.

Optimizations of the maize model with an added N_2_-fixing bacterial symbiont were performed as in Holland et al. (Holland et al. 2023). Briefly, the plant model without the bacterial symbiont is first optimized to maximize RGR and the ammonium uptake necessary to support this maximized growth rate is recorded. This ammonium uptake rate defines the point at which the model's biomass accumulation is no longer limited by ammonium availability. For both the model with and without the associated N_2-_fixing symbiont, we then continuously decrease the external ammonium level and evaluate the RGR at each point. We also calculate the “grams of carbon per gram nitrogen,” or g C g^−1^ N, which is a measure of the ratio of carbon, in the form of glucose or fatty acids, sent to the fungus from the plant in exchange for nitrogen from the fungus. g C g^−1^ N was calculated as in Holland et al. (Holland et al. 2023):


(6) $$\begin{array}{c} C\;:N\;\mathrm{ratio}=\frac{V_{c}}{2\;V_{n}} \end{array}$$

where *V_c_* is the rate of CO_2_ release from the nodule tissue and V_n_ is the flux through the bacteroid nitrogenase reaction.

### AMF model optimization and analysis

The AMF model was optimized in a sequential process meant to mathematically encode the following assumptions about the plant/AMF symbiosis:

1. Since the AMF fungus is dependent on carbon from the host plant, the ability of the fungus to confer nutrient uptake benefits to the plant is a function of carbon investment from the plant.
2. To achieve a measured level of nutrient uptake benefits because of AMF inoculation, the modeled system must achieve the same ratio of AMF to plant biomass, with decreasing ratios of AMF to plant biomass linearly decreasing the nutrient uptake benefits conferred.

The data for phosphorus uptake benefits in AMF- and AMF + plants (Sawers et al. 2017), data for nitrogen uptake benefits (Valentine et al. 2001), and HLD (Sawers et al. 2017) values were used to estimate the AMF biomass necessary to achieve maximal nutrient uptake benefits. We estimated the dry weight of AMF tissue $\left(DW_{AMF}\right)$ from the HLD values and assuming the plants were grown in 2,750 grams of soil (Sawers et al. 2017), as described in Equation E7.


(7) $$\begin{array}{c} DW_{AMF}=\left(\pi r^{2}\left(HLD_{low\;P}\left(\frac{m\;\mathrm{hyphae}}{g\;\mathrm{soil}}\right)\times 2750g\;\mathrm{soil}\right)\right)\\ \left(\frac{1.09\times 10^{6}\;\mu g\;\mathrm{hyphae}\;FW}{\mathrm{hyphae}\;cm^{3}}\right)\left(\frac{0.21\mu g\;hyphae\;DW}{\mu g\;\;hyphae\;FW}\right)\left(\frac{9.75\mu g\;fungal\;DW}{\mu g\;hyphae\;DW}\right) \end{array}$$

where *HLD*_low P_ is the hyphal length density in centimeters reported in the lowest phosphorus condition. The conversion between hyphae fresh weight (FW) and hyphae volume, $\left(\frac{0.21\mu g\;hyphae\;FW}{\mathrm{hyphae}\;cm^{3}}\right)$, is taken from literature estimates, as is the fresh weight to dry weight (DW) conversion (Bakken and Olsen 1983). Finally, given that substantial fungal biomass is contained in non-hyphal tissue like vesicles, we used a reported ratio of hyphal to total (hyphal plus vesicle) biomass (Olsson and Johansen 2000), $\left(\frac{9.75\mu g\;fungal\;DW}{\mu g\;hyphae\;DW}\right)$, to convert from the hyphal biomass estimate to an estimate of total fungal dry mass.

To optimize the system, the maximal biomass growth of the system without any AMF-mediated nutrient uptake benefits is calculated across a range of phosphate and ammonium uptake rates as described in Eq. 4–5.

The ratio of the AMF- plants’ biomass and the AMF itself in the low-phosphorus condition reported in Sawers et al. (Sawers et al., 2017) was then used to define the following reactions in the model:


(8) $$\begin{array}{c} (\mathrm{Growt}h_{in})\mathrm{Biomas}s_{AMF}\times AMF_{\mathrm{proportion}}\to P_{\mathrm{uptake}}+N_{\mathrm{uptake}} \end{array}$$

(9) $$\begin{array}{c} (2.16\times P_{n})\mathrm{Orthophosphat}e_{\mathrm{fungus}}+P_{\mathrm{uptake}}\to (2.16\times P_{n})\mathrm{Orthophosphat}e_{\mathrm{plant}} \end{array}$$

(10) $$\begin{array}{c} (0.23\times N_{i})\mathrm{Ammoniu}m_{\mathrm{fungus}}+N_{\mathrm{uptake}}\to (0.23\times N_{i})\mathrm{Ammoniu}m_{\mathrm{plant}} \end{array}$$

where *Growth_in_* is the maximal biomass accumulation that the plant is capable of in the absence of AMF-mediated nutrient uptake benefits, AMF_proportion_ is the proportion of AMF-to-plant biomass, and *P_n_* and *N_i_* are the non-AMF-mediated plant phosphate and ammonium uptake rates. A single unit of flux through this reaction requires producing the observed amount of fungal biomass relative to plant biomass, yielding a single unit of *P_uptake_* and *N_uptake_*, which can be used in Eq. 9 and Eq. 10 to move 2.16 times the basal phosphate uptake rate or 0.23 times the basal ammonium uptake rate. The upper bound of Eq. 8 is set to 1, such that no more than 2.16 times the basal phosphate uptake rate and 0.23 times the ammonium uptake rate can be achieved, based on reported nitrogen and phosphate uptake benefits (Valentine et al. 2001; Sawers et al. 2017).

With these additions, the full model, plant, and AMF partner, is optimized as described in Eq. 4–5. In addition to the maximal RGR of the plant, the fluxes of glucose and palmitate from the plant to the fungus and the net CO_2_ uptake of the plant are recorded. From these quantities, we calculate the percent of CO_2_ from the plant that is invested in the AMF partner:


(11) $$\begin{array}{c} \text{\%}\;\mathrm{Carbon}\;\mathrm{Allocation}=(100)\frac{v_{CO_{2}\mathrm{uptake}}}{\left(16\;v_{\mathrm{palmitate}})+(6\;v_{\mathrm{glucose}}\right)} \end{array}$$

where *v*_CO2 uptake_ is the plant's CO_2_ uptake flux, and *v*_palmitate_ and *v*_glucose_ are the fluxes of palmitate and glucose transport from the plant to the fungus.

### AMF growth rate validation experimental setup

To generate AMF-incompatible (AMF-) maize hybrids for field evaluation, a B73 inbred line homozygous for the *castor-2* mutation (Ramírez-Flores et al. 2020) was crossed as male to a W22 inbred line also homozygous for *castor-2*. Control AMF-compatible (AMF+) hybrids were generated by crossing the same B73 *castor-2* line as male to an AMF- W22 inbred line carrying a mutation in the maize ortholog of the *pollux* gene (Gutjahr et al. 2008; Ramirez-Flores 2015), with trans-complementation recovering AMF compatibility in the F_1_ hybrid progeny. This scheme allowed that both AMF- and AMF + hybrids were produced from AMF- mothers to reduce any strong maternal effect.

AMF + and AMF- maize hybrids were evaluated at Russell E. Larson Agricultural Research Center at the Pennsylvania State University (40°42′39.1″N 77°57′11.4″W) within a larger split-split-plot design with 2.5-foot row spacing receiving drip irrigation. Prior to planting, the soil of 90 random rows (14.1 m^2^ density) was sampled by pooling soil from the front, middle, and end of rows at a depth of 0–10 cm. Soil samples were then submitted to the Agricultural Analytical Services Lab to determine Total N and Mehlich 3 extractable P by ICP analysis, with soil parameters at unsampled rows modeled with kriging interpolation using the “gstat’ R package(Gräler et al. 2016). Seeds were planted on 18 May 2023, 1 wk after 150 lbs of 19–19–19 (%N−%P−%K) fertilizer was applied. After receiving an additional 300 lbs of urea per acre on 21 June 2023, AMF + and AMF- plant biomass was sampled, dried, ground, and analyzed with a dry combustion elemental analyzer to determine Total N content (as described in (White et al. 2021)) at timepoints T1-T4 (28, 38, 48, and 58 days after emergence) and at harvest on 4 October 2023. To assay AMF colonization in AMF + (32% colonization; n = 117) and AMF− (2% average colonization, n = 57) plants, root tissue was harvested at 8-wk after emergence to quantify fungal colonization via microscopy following staining. Briefly, ∼0.25 g of fine root tissue was collected from 0–0.25 m depth, cleared in 10% KOH at 70 °C over 48 h, rinsed 5 times in deionized water, incubated in 0.3 M HCl for 15 mins at room temperature, and stained at 96 °C for 8 min using a 0.05% w/v Direct Blue 15 Dye staining solution in a 1:1:1 mixture of lactic acid: glycerol: deionized water. Percent root length colonized was then determined by noting the presence and absence of AMF structures at 40 × magnification along 100 intersections on a 1 cm x 1 cm grid (GIOVANNETTI and MOSSE, 1980).

### Soil N and P estimation

Available N in the field trial was estimated by adding the known N application rate to an estimate of N mineralization from soil organic matter. Soil organic matter (SOM) content was estimated from measured total N, assuming a standard composition of SOM of 5% nitrogen (Pribyl 2010; Paul 2016). From this, we calculate the contribution of mineralized organic nitrogen as:


(12) $$\left(\frac{2.48e6\mathrm{kg}\;\mathrm{soil}}{\mathrm{ha}}\right)\left(\frac{2.2\mathrm{kg}\;SOM}{100\mathrm{kg}\;\mathrm{soil}}\right)\left(\frac{5\mathrm{kg}\;N}{100\mathrm{kg}\;\mathrm{SOM}}\right)\left(\frac{2.0\mathrm{kg}\;\mathrm{SOM}\;\mathrm{mineralized}}{100\mathrm{kg}\;\mathrm{SOM}}\right)=54.56\;\frac{\mathrm{kg}\;N}{\mathrm{ha}}$$

where the 2.2 kg SOM 100 kg^−1^ soil is derived from the measured total N, the weight of a hectare of soil is estimated assuming a bulk density of 1.24 g/cm^3^ in the 0–20 cm depth (Panagos et al. 2024), SOM is assumed to be 5% N by weight (Pribyl 2010; Paul 2016) and 2.0 kg of SOM is assumed to be mineralized per 100 kg of SOM per growing season (Sullivan et al. 2020). The 0–20 cm depth is used for the mineralization calculation because the majority (∼80%) of the root surface area of a maize plant is present between soil depths of 0–20 cm (Zhou et al. 2019).

The average soil phosphorus level across all planted rows was approximately 59 mg kg^−1,^ and the average total nitrogen content was 0.11% (kg N kg^−1^ soil) (Dataset S3). A previous study (Sawers et al. 2017) showed that both shoot dry weight gains and AMF-mediated growth benefits were previously shown to disappear somewhere between phosphorus concentrations of 15.5 mg kg^−1^ and 53.2 mg kg^−1^. This indicates that the plants in this study had adequate phosphorus available to achieve optimal or near-optimal growth. Corroborating this idea, one study found that high-performing maize lines take up an average of 114 kg P_2_O_5_, or 50 kg P, during the growing season (Bender et al. 2013), as compared to the 66 kg P that were available to a depth of 20 cm in the samples with the lowest soil P concentration in this study (26.5 mg/kg). This means that the plants assessed in this study received 100%+ of the P necessary to achieve optimal growth. The average total nitrogen content in our plots of 0.11% was measured prior to an application of 187 kg N ha^−1^. Together, the mineralization of the soil organic matter and the applied N fertilizer amount to the equivalent of an input of 241 kg N ha^−1^. Assuming that our maize hybrids can achieve similar optimal growth rates as those reported in a previous study assessing optimal N application rates and nutrient uptake rates (Bender et al. 2013), high-performing maize hybrids will take up an average of 286 kg N ha^−1^. This means that the plants assessed in this study received ∼84% of the N necessary to achieve optimal growth. Together with the P availability, this makes the nitrogen-limiting upper-right-hand quadrant the appropriate comparison between the RGR improvements in Fig. 1a and our empirical measurements.

### Plant RGR estimation

The RGR of plants from the field trial dataset was estimated using the following equation:


(13) $$\begin{array}{c} RGR=\frac{\ln\;(DW_{1})-\ln\;(DW_{2})\;}{t_{2}-t_{1}} \end{array}$$

where *DW_1_* and *DW_2_* are the shoot dry weight measurements (g) at growth timepoints 1 (*t_1_*) and timepoint 2 (*t_2_*), which are 28 and 38 days after emergence, respectively. These 2 early growth timepoints were used to calculate RGR to minimize any potential impact of differing developmental trajectories between AMF + and AMF—plants.

### Correction for changes in biomass P due to AMF status

To account for changes in biomass P content between AMF- and AMF + plants, an additional reaction was added to AMF + models that consume orthophosphate at a rate proportional to biomass accumulation. This rate was 0.029 mmol gDW^−1^ for the comparison between model predictions and the field data gathered in N-limited conditions, and 0.019 mmol gDW^−1^ for the comparison with the P-limited growth data reported in (Sawers et al. 2017). This was implemented by adding a ratio constraint using the *addRatioReaction()* function in the COBRA Toolbox (Heirendt et al. 2019).

### Sensitivity analysis

Local sensitivity analysis was performed by varying the values of key input parameters for all models presented in this study by ±10% and then calculating the percent change in (i) RGR, (ii) g C g^−1^ N, and (iii) g C g^−1^ P. The reported sensitivity values are the averages of the percent changes resulting from increasing and decreasing a given parameter value.

### Monte Carlo estimation of uncertainty

A Monte Carlo approach was implemented to calculate the distributions of RGR benefits and carbon allocation percentages from the plant-AMF model. The uncertainty of the AMF-mediated P uptake benefits and the necessary AMF biomass was estimated from (Bakken and Olsen 1983; Sawers et al. 2017). Uncertainty data were not available for the AMF-mediated N uptake benefits, so the coefficient of variation of this parameter was assumed to be the same as that of the P uptake benefit. Monte Carlo samples were generated by varying these 3 parameters according to their means and standard deviations (Table S1), and the resulting RGR and carbon allocation estimates were used to generate confidence intervals. Monte Carlo samples (between 400 and 1,100, depending on the analysis performed) were generated in sets of 100 simulations, and sampling was stopped once the upper and lower bounds of the 95% CIs were observed not to change more than 0.1% relative to their previously calculated value.

### Software

Optimizations and model analysis were performed in MATLAB R2020a using the COBRA Toolbox 3.0 (Heirendt et al. 2019), the Enterprise IBM ILOG CPLEX solver version 12.10, and the Gurobi Optimizer 11.02. Code to run the model and reproduce the manuscript results can be found at: https://github.com/Matthews-Research-Group/Maize-Rhizobia-AMF_FBA.

## Supplementary Material

kiag327_Supplementary_Data

## Data Availability

All data, models, and code needed to reproduce the analysis in this study can be found in the online supplemental material accompanying the manuscript and at https://github.com/Matthews-Research-Group/Maize-Rhizobia-AMF_FBA.
